# *Mycobacterium tuberculosis* functional genetic diversity, altered drug sensitivity, and precision medicine

**DOI:** 10.3389/fcimb.2022.1007958

**Published:** 2022-10-03

**Authors:** Sydney Stanley, Qingyun Liu, Sarah M. Fortune

**Affiliations:** Department of Immunology and Infectious Diseases, Harvard T.H. Chan School of Public Health, Boston, MA, United States

**Keywords:** *mycobacterium tuberculosis*, antibiotic resistance, genetic diversity, precision medicine, bacterial genomics, molecular diagnostics, TB drug regimen, MIC

## Abstract

In the face of the unrelenting global burden of tuberculosis (TB), antibiotics remain our most effective tools to save lives and control the spread of *Mycobacterium tuberculosis* (Mtb). However, we confront a dual challenge in our use of antibiotics: simplifying and shortening the TB drug regimen while also limiting the emergence and propagation of antibiotic resistance. This task is now more feasible due to the increasing availability of bacterial genomic data at or near the point of care. These resources create an opportunity to envision how integration of bacterial genetic determinants of antibiotic response into treatment algorithms might transform TB care. Historically, Mtb drug resistance studies focused on mutations in genes encoding antibiotic targets and the resulting increases in the minimal inhibitory concentrations (MICs) above a breakpoint value. But recent progress in elucidating the effects of functional genetic diversity in Mtb has revealed various genetic loci that are associated with drug phenotypes such as low-level MIC increases and tolerance which predict the development of resistance and treatment failure. As a result, we are now poised to advance precision medicine approaches in TB treatment. By incorporating information regarding Mtb genetic characteristics into the development of drug regimens, clinical care which tailors antibiotic treatment to maximize the likelihood of success has come into reach.

## Introduction

As the tuberculosis (TB) pandemic caused by *Mycobacterium tuberculosis* (Mtb) rages on, we must find new ways to augment the efficacy of antibiotics, our most powerful weapons in this war (The World Health Organization, 2021). Precision medicine represents a promising strategy, which would involve methods to mitigate the occurrence of treatment failure by predicting the antibiotic sensitivities of individual strains based on their genotype and modifying the drug regimen accordingly (Lange et al., 2020). Adopting precision medicine approaches such as tailored antibiotic treatment regimens informed by the results of next-generation molecular detection of mutations known to alter drug susceptibility could facilitate shortened treatment regimens and mitigate the development of resistance (Lange et al., 2020). However, such strategies require a roadmap of the relationship between bacterial variation, antibiotic sensitivities, and treatment outcomes. In this review, we will discuss advancements in our understanding of how Mtb genetic diversity affects antibiotic susceptibility, which has expanded conventional metrics and measures of drug sensitivity. We will highlight opportunities to apply this knowledge to bolster current efforts towards precision medicine for TB.

## The genesis of our current understanding of drug resistance

The challenge of drug resistance in Mtb became clear almost immediately after the discovery of streptomycin (SM) in 1943 (Schatz et al., 1944). By 1947 two randomized controlled trials had been conducted to assess the efficacy of SM for TB (Marshall et al., 1948; Fox et al., 1954; Fox et al., 1999). In these trials, there was no long-term survival benefit to SM treatment; almost all patients receiving SM initially improved but most subsequently relapsed with drug resistant Mtb. These findings led to the recognition that Mtb with reduced antibiotic susceptibility would reliably emerge after a period of antibiotic selection, at least with a single agent.

In the late 1940’s and early 1950’s, drug susceptibility was measured by comparing the lowest concentration of a drug that inhibited the *in vitro* growth of strains isolated from patients to that of a reference strain, H37Rv, which had been derived from a lung lesion 50 years earlier (Mitchison, 1949). The presumption was that most Mtb strains at the time were drug sensitive by definition because they had not faced drug treatment before. More formally, the diagnostic assumption was that bacterial growth distributions in the presence of drug were normally distributed, and that all strains falling above this normal distribution were equally likely to fail SM treatment. This understanding simplified the technical complexity of standardizing measurements of drug responses and led the field to solutions for establishing resistance, such as the calibrated “minimum inhibitory concentration” (MIC), which was a comparative measure response in strains deemed “probably sensitive” (from newly diagnosed patients) and “probably resistant” (from treatment failures) (Fox et al., 1999). These approaches have now matured into the so-called proportion method whereby the critical concentration is the lowest concentration of drug, established by international convention, which inhibits 99% of the growth of a population of “phenotypically wild type” Mtb (The World Health Organization, 2018). The MIC is now defined as the lowest concentration of an antibiotic that inhibits visible bacterial growth of an individual strain and is often used by bench scientists (Schön et al., 2020). The MBC or minimum bactericidal concentration is the lowest concentration that kills 99% of the initial bacterial population and is utilized mostly in clinical settings (National Committee for Clinical Laboratory Standards, 1999). Even though the field recognizes a difference between low and high resistance to some antibiotics such as isoniazid (INH) or fluoroquinolones (Malik et al., 2012; Lempens et al., 2018), proportion methods such as these - or genetic proxies of proportion methods - remain the most commonly used approach to defining drug susceptibility in Mtb, and has had the effect of enforcing a dichotomized understanding of antibiotic resistance, where the “wildtype” is assumed to be more or less homogeneously sensitive and functionally drug naïve.

We note that that the assumption that “wildtype” Mtb is drug naïve was challenged early on by the increasing frequency of primary drug resistance, that is drug resistance in treatment naïve patients. By 1964, not even 20 years after the first SM trial, 11% of primary TB cases in Hong Kong were drug resistant (and 10% resistant to INH and/or SM in Kenya by 1974), reflecting remarkable drug pressure on the extant bacterial population (Hong Kong Government Tuberculosis ServiceBritish Medical Research Council Co-operative Investigation, 1964; An East African and British Medical Research Council Co-operative Investigation, 1978) even as measured by dichotomous assays. We propose that it is likely that the population distribution of other forms of altered drug susceptibility, not captured by conventional measures such as MIC assays and therefore not countered through subsequent regimen modifications, were also under this immense selective pressure and increasingly prevalent in the population.

Nonetheless, in a pragmatic field, the dichotomous definition of resistance became accepted as adequate where there was little capacity to measure drug susceptibility or tailor therapy based on bacterial features. Thus, as the modern short course chemotherapy regimens were developed through the 1980’s, length of treatment was established in part to minimize the effects of preexisting bacterial or host differences in drug responses that contributed to treatment failure. Radically simpler regimens were first tried in the 1950s - 3 months of INH alone, for example, which actually led to durable cure in nearly half of patients (46% or 62/134) (Blowers and Cooke, 1954), but the focus was on developing a universal regimen because there was little capacity to identify and act on predictors of short course therapy success or failure.

Given that drug regimens are designed around dichotomized measures of Mtb antibiotic resistance, in the genomic age the field has focused on delineating the bacterial genetic determinants that mediate MIC shifts above the critical concentration resistance breakpoint. This resulted in studies that identified and characterized antibiotic resistance determinants by zeroing in on individual mutations in the genes encoding drug targets or drug activators, such as single nucleotide polymorphisms in *katG* and *inhA*, *rpoB*, *embB*, *pncA*, and *rpsL* and *rrs* conferring resistance to INH, RIF, pyrazinamide (PZA), ethambutol (EMB), and SM respectively (Finken et al., 1993; Scorpio et al., 1997; Sreevatsan et al., 1997; Rozwarski et al., 1998; Ramaswamy et al., 2003; Hazbón et al., 2006; Juréen et al., 2008; Palomino and Martin, 2014). Such studies informed the development of GeneXpert MTB/RIF (Cepheid, Sunnyvale, CA, USA), the first test designed for rapid molecular detection of Mtb and RIF resistance (Boehme et al., 2010). Later advancements include Xpert MTB/XDR, which completes rapid susceptibility testing for additional first- and second-line drugs to partially address multidrug-resistant (MDR) and extensively drug-resistant (XDR) TB (Cao et al., 2021; Penn-Nicholson et al., 2022). These tests and other accessible rapid diagnostics represent a transformative first-step towards TB precision medicine. However, there is considerable space for improvement because drug resistance-conferring mutations and breakpoint MICs are not completely predictive of treatment outcomes (Liu et al., 2021). Therefore, the field has begun to consider the wider effect of Mtb genetic diversity on not only antibiotic resistance but other clinically-relevant antibiotic susceptibility phenotypes.

## Moving beyond dichotomous measures of drug resistance

While the clinical TB field was focused on acquired, high-level drug resistance determined by MIC changes above a breakpoint value, the bench sciences have been rapidly expanding our understanding of the clinical implications of variation in MIC below the critical concentration and the associated phenotypes. This includes small increases in MIC below the resistance threshold and also tolerance and persistence phenotypes that have been reviewed previously (Lewis and Shan, 2017; Boldrin et al., 2020; Schrader et al., 2020). These also include a range of environmentally-driven alterations in drug susceptibility such as differentially culturable bacteria (Chengalroyen et al., 2016; Turapov et al., 2016), “fat and lazy” macrophage resident bacteria (Garton et al., 2008; Daniel et al., 2011), or bacterial subpopulations created by adaptive regulatory systems like the toxin-antitoxin systems (Slayden et al., 2018).

Historically, there has been little effort to incorporate these other forms of “drug conditioning” into clinical treatment frameworks because they have been somewhere between hard and impossible to measure in a clinical setting and of unclear clinical importance. However, there is data to suggest that we should pay attention to the spectrum of altered drug responses under high-level drug resistance, the mass of ice under the tip of the iceberg.

MIC measurements are accessible to clinical labs. Indeed, a range of MIC levels for Mtb clinical isolates were observed as early as 1953 when the methodology for the MIC determination of INH, SM, and para-aminosalicylic acid (PAS) was becoming standardized, but this was assumed part of the “wildtype”, drug sensitive distribution (Medical Research Council, 1953). However, in a landmark study, Colangeli et al. determined that putatively drug susceptible Mtb strains with higher MICs to RIF and INH are more likely to result in treatment failure (Colangeli et al., 2018). This observation indicates that small increases in MIC are clinically-relevant even though they have been historically overlooked and suggest a potential benefit of having the tools to identify these features.

With the advent of inexpensive sequencing technology, bacterial genomic data has also become widely available. This has been leveraged by methods such as candidate gene approaches to determine if mutations in known target genes of first-line drugs predict phenotypic antibiotic susceptibilities and bacterial genome-wide association studies (GWAS) to identify bacterial genetic variations associated with high-level drug resistance (Zhang et al., 2013; Coll et al., 2018; CRyPTIC consortium and the 100,000 genomes project, 2018; Hicks et al., 2018; Farhat et al., 2019; Lai and Ioerger, 2020). While the latter were originally undertaken to identify missing genetic determinants of MICs above breakpoint values, these studies have unearthed a suite of “stepping stone” mutations (Hicks et al., 2018; Safi et al., 2019; Hicks et al., 2020; Liu et al., 2022; Martini et al., 2022). These are mutations that at a minimum facilitate the acquisition of drug resistance but presumptively this occurs by reducing treatment efficacy. There have been few treatment cohorts with good metadata sufficiently powered to identify these factors through their direct association with treatment outcomes. However, the population genomic argument is that the selection in the population is de facto evidence of their benefit to the bacterium. We would argue that these bacterial factors will become even more important as we push to shorten treatment, though relative importance of specific factors may differ by regimen (Turkova et al., 2022).

The ultimate importance of identifying and parsing the genetic basis of these other forms of drug susceptibilities depends on the implications of and ability to act on this knowledge. We would argue that critical factors include (A) ability to modify drug regimen based on this information and (B) implications for new drugs – that is, mutations that do not alter drug responses in a target-specific fashion but broadly alter responses in a way that will compromise new regimens.

## Non-canonical bacterial genetical determinants and low-level MIC shifts

The genetic basis of the low-level INH and RIF MIC shifts associated with treatment failure by Colangeli et al. was not defined. However, several studies have suggested a range of candidate variants. These include mutations in both known pathway genes and novel pathways. Mutations associated with antibiotic resistance differ in how much they modulate MIC, for example the *katG* S315T mutation is associated with large, resistance-level increases in the MIC of INH, while other mutations such as the *fabG1* C-15T polymorphism upstream of the INH target *inhA* results in smaller MIC shifts and low-level resistance (Lavender et al., 2005; Palomino and Martin, 2014). As for non-canonical targets, a Lineage 1 (L1) subclade-defining mutation in *ndh* (R268H) was associated with a ~2-fold increase in INH MIC, deemed neutral by the authors but only because it did not meet a standardized definition of resistance despite being present in 9.5% of INH-resistant strains but in none of the susceptible isolates, consistent with the changes in drug susceptibility associated with treatment failure (Lee et al., 2001; Merker et al., 2020).

Additionally, non-target variants associated with drug resistance that may contribute to moderate yet clinically-relevant MIC changes were identified by bacterial population GWAS studies (Zhang et al., 2013; Coll et al., 2018; Hicks et al., 2018; Farhat et al., 2019; Lai and Ioerger, 2020). Mutations in *dnaA*, which are found in 3.2% of all clinical strains globally, have been linked to INH resistance and have ~2-fold increase in MIC (Hicks et al., 2020). Low-level INH MIC-shifts may be relevant for new and old regimens alike, where the new 4-month regimen (rifapentine, moxifloxacin) still contains an INH backbone (Dorman et al., 2021). Further, *rnaseJ* is highly mutated in drug-resistant clinical strains of Mtb (Martini et al., 2022). While deletion of the gene does not alter the MIC to RIF nor INH, it does increase multidrug tolerance (Martini et al., 2022). Mutations in *resR* (Rv1830), a gene undergoing positive selection in Mtb clinical isolates, result in a slight MIC increase to INH, but more interestingly leads to faster bacterial recovery from drug treatment (Liu et al., 2022). ResR belongs to a regulatory pathway with WhiB2 and WhiA, and mutations in these genes were associated with canonical drug resistance and relapse after antibiotic treatment (Liu et al., 2022). Around 1.5%-9.7% of drug-sensitive Mtb strains from high-burden TB countries carry mutations in these genes, but mutations are present in 22.2% of strains from patients who failed treatment in the global REMoxTB phase 3 regimen-shortening trial (Bryant et al., 2013; Jindani et al., 2014; Liu et al., 2022).

Low-level MIC shifts have also been observed for second-line drugs. Mutations in *gidB* are associated with low-level SM resistance, but patients infected with Mtb strains carrying the *rrs* mutation that confers high-level amikacin resistance may still benefit from SM treatment despite the presence of *gidB* variants (Spies et al., 2011; Wong et al., 2011; Cohen et al., 2015; Cohen et al., 2020). *gidB* mutation may also explain the 15% of SM-resistant Mtb strains that lack mutations in *rrs* and *rpsL* (Wong et al., 2011). Even though SM is no longer used in the treatment of TB, this example illustrates the diagnostic benefit of non-target genes that mediate low-level resistance (Cohen et al., 2020). Mutations that impair the monooxygenase *Rv0565c* are associated with low-level resistance to the second-line antibiotic ethionamide (ETH) (Hicks et al., 2019). Like loss-of-function (LOF) mutations in *ald* that result in D-cycloserine resistance, Rv0565c mutations are found exclusively in MDR strains of Mtb, suggesting selection by second-line drug regimens (Desjardins et al., 2016; Hicks et al., 2019). Interestingly, low-level resistance has also been described for BDQ and CFZ, mediated by mutations in non-target genes including the regulator of the MmpS5-MmpL5 efflux pump system and *pepQ* (Andries et al., 2014; Almeida et al., 2016), and is more prevalent than predicted by exposure to these drugs.

## Mtb genetic variation and antibiotic susceptibility

The original framing of altered drug susceptibility in Mtb implicitly posited that there were not clinically relevant differences in antibiotic susceptibility between Mtb strains before antibiotic exposure. However, as the L1 subclade-defining mutation in *ndh* suggests, it has become clear that this is an oversimplified view (Oppong et al., 2019; Merker et al., 2020).

Numerous studies reviewed elsewhere have indicated that L2 and L4 strains are more associated with drug resistance, MDR, and XDR (Gygli et al., 2017; Shanmugam et al., 2022). Several studies have demonstrated that L2 Mtb strains acquire drug resistances more rapidly *in vitro* (Ford et al., 2013), and are also more likely to develop RIF resistance after becoming resistant to INH (Torres Ortiz et al., 2021). This may reflect factors such as different mutation rates or epistatic interactions with lineage- or sublineage-associated mutations (Borrell and Gagneux, 2011; Ford et al., 2013; Torres Ortiz et al., 2021).

Other epistatic effects have been described. Fenner et al. discovered that compared to other lineages, L1 strains are more likely to carry a specific a promoter mutation in *inhA*, a drug resistance determinant that encodes the enzymatic target of INH (Fenner et al., 2012). In addition, Mtb genetic background modulates the level of INH resistance conferred by other mutations known to mediate resistance, which suggests an epistatic interaction between drug resistance mutations and lineage genetic diversity (Fenner et al., 2012). Similarly, the essentiality of *katG*, the activator of INH, was shown to differ depending on strain genetic background (Carey et al., 2018). Further, the degree of essentiality for *katG* is associated with the *katG* mutation rate, suggesting that the epistatic interaction between *katG* essentiality and strain genetic background is associated with the development of drug resistance (Carey et al., 2018).

Torres et al. identified “pre-resistance” genomic loci and polymorphisms associated with increased risk for drug resistance acquisition which could inform precision medicine efforts to predict and preempt the occurrence (Torres Ortiz et al., 2021). Following the idea of pre-resistance, a subset of L2 strains carry an ancestral polymorphism in *gidB*, which could mediate some level of intrinsic SM resistance (Spies et al., 2011). Additionally, a subgroup of MDR L4 strains share a mutation in *tlyA*, a gene that mediates resistance to the second-line drug capreomycin (Walker et al., 2018; Merker et al., 2020). And fortunately, altered susceptibility mediated by clade-defining mutations is not always bad news. For example, a LOF mutation in *whib7* shared by a subgroup of L1 strains results in increased macrolide sensitivity (Li et al., 2022). This subgroup is estimated to cause 43,000 cases of MDR-TB per year (Li et al., 2022; Edokimov et al., 2022). Further, specific clades of L1 and L4 carry LOF mutations in *mmpl5* that could render them hypersusceptible to bedaquiline (BDQ) and clofazimine (CFZ) (Andries et al., 2014; Merker et al., 2020). Given the geographical distributions of Mtb lineage subgroups, these data underscore the need for drug susceptibility diagnostics that detect variants associated with the regional context (Manson et al., 2017).

## Host-pathogen interactions shaping Mtb drug responses

In the cases of lineage or sublineage defining variants in genes like *whib7* or *mmpl5*, Mtb strains have acquired altered sensitivity to drugs they have not seen before. This is often attributed to genetic drift. However, the alternative model is that there are some host environments which select for bacterial features that that are also advantageous to Mtb in the face of drug (Hicks et al., 2018; Safi et al., 2019). By identifying variants that alter Mtb antibiotic susceptibility in host-relevant contexts, the field has diversified the suite of candidate mutations that could improve molecular diagnostics.

Several studies have linked Mtb antibiotic sensitivity to features of the host environment such as carbon source availability. For example, Mtb glycerol starvation triggers a stress-resistance response that in turn promote multidrug antibiotic tolerance (Safi et al., 2019). This phenotype is mediated by transient frameshift mutations that impair the glycerol kinase-encoding gene *glpK*, mutations in which are associated with MDR and XDR Mtb strains (Bellerose et al., 2019; Safi et al., 2019). Nonsynonmoyous *glpK* mutations were identified in 6.6% of Mtb strains from a Peruvian cohort (Bellerose et al., 2019). Mutations in *glpK* accrue during *in vitro* drug treatment and human infection, so this serves as another example of how bacterial adaptation to the host may augment antibiotic susceptibility (Bellerose et al., 2019; Safi et al., 2019; Vargas and Farhat, 2020). Mutations in the transcription factor encoded by *prpR* are associated with drug resistant Mtb clinical strains (Hicks et al., 2018). *prpR* mutations mediate drug tolerance to INH, RIF, and the second-line antibiotic ofloxacin (OFL) only during macrophage infection or in liquid media supplemented with propionate (Hicks et al., 2018). *prpR* mutations are enriched in Chinese clinical isolates, with a prevalence of 8-10% (Hicks et al., 2018). The glycerol and propionate phenotypes are consistent with other studies that investigated Mtb drug tolerance and phosphoenolpyruvate starvation in the context of the nonreplicating state induced by hypoxia, a stress relevant to the lung environment that selects for resistance (Liu et al., 2016; Lim et al., 2021). Similarly, Dhar and McKinney discovered that *cydC* and the rv0096–rv0101 gene set are persistence genetic determinants that alter Mtb clearance in C57BL/6 mice treated with INH (Dhar and McKinney, 2010). The persistence phenotypes are dependent on the mouse tissue environment, suggesting an interaction between the Mtb physiological changes caused by mutations in these genes, INH, and components of the mouse tissue (Dhar and McKinney, 2010).

Bacterial variants and differences in host environments are likely to be compounded by host differences in the pharmacokinetics and pharmacodynamics of drug metabolism. It is clear that drug exposure matters. RIF concentrations 2 hours post-dosing is associated with TB treatment success (Ramachandran et al., 2017). Further, Chigutsa et al. determined that Mtb infection sterilization can be predicted by a nonlinear relationship between patient antibiotic concentrations and bacterial MICs (Chigutsa et al., 2015). Indeed, data suggest that altered susceptibility indicated by strain associated differences in MIC can be overcome with increased dosing (Ruesen et al., 2018).

## Conclusion and discussion

**Figure 1:**
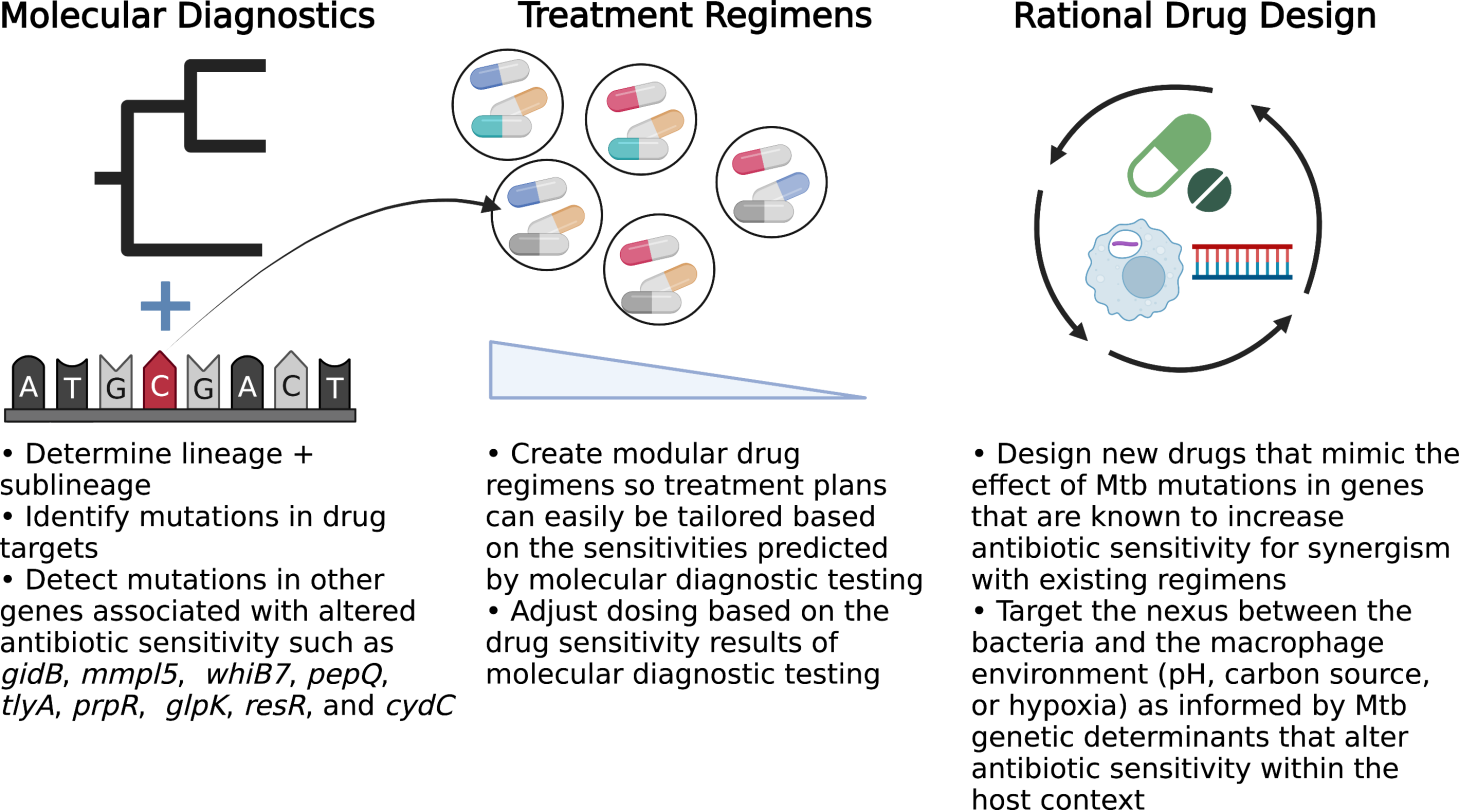
Opportunities to implement precision medicine approaches to improve TB treatment. BioRender.com.

Traditionally, Mtb antibiotic response studies focused on bacterial mutations in genes involved in the drug mechanism of action and MIC shifts above the resistance threshold. But examination of the larger role of Mtb genetic diversity and the associated clinically-relevant drug phenotypes has unearthed new avenues that can be exploited to develop the precision medicine toolkit and ultimately improve patient outcomes (Cohen et al., 2019) (**Figure 1**). By expanding the scope of molecular diagnostic tests, we can identify mutations in strains that allow us to predict antibiotic sensitivities and treatment failure, which direct us in optimizing the drug regimen and even dosing to maximize the likelihood of treatment success.

We recognize that there are barriers to precision medicine in TB, including the cost of developing and implementing new tools and also epistatic interactions between diagnostic Mtb mutations and bacterial and host factors that could alter the phenotype penetrance and predictive power across populations. Therefore, we propose further research into understanding the prevalence and penetrance of potentially diagnostic mutations. This necessitates more studies that collect treatment outcome data and also WGS Mtb isolates. It is true that individual mutations may only have the power to predict resistance in a portion of strains, for example *gyrA* mutations only occur in 50-90% of fluoroquinolone-resistant clinical Mtb isolates (Brossier et al., 2010; Yin and Yu, 2010; Singh et al., 2015). To cover the lower end of the range, we should consider finding suites of mutations that together highly associate with resistance or clinical outcomes, a method that has already shown to be effective (Walker et al., 2015).

We can start by exploring mutations noted in a 2021 WHO catalogue of mutations associated with drug resistance, but in order to improve upon the growing collection of Mtb mutations with diagnostic potential, we should diversify our repertoire of phenotypes of clinical interest beyond just MIC (Walker et al., 2022). For example, further exploration into prevalent mutations in Mtb clinical isolates that modulate MBC, rather than just MIC, could reveal interesting predictors of host-relevant antibiotic tolerance phenotypes (Kalia et al., 2017; Sarathy et al., 2018; Dutta et al., 2019; Kreutzfeldt et al., 2022). Further, we should integrate Mtb strain characteristics with information regarding patient factors such as genetics, metabolism, environment, and geographic location to find novel ways to optimize treatment regimens and dosing. Given the presence of sublineage-defining mutations in genes associated with resistance and the endemic nature of these strains, even-high level patient information such as country or region of origin could inform which antibiotics may confer the maximum benefit (Manson et al., 2017; Walker et al., 2018; Hicks et al., 2020; Merker et al., 2020; Li et al., 2022). In the future, exploiting what we know and what we discover regarding host-pathogen interactions and altered antibiotic sensitivity can inform rational drug and rational drug regimen design. But in the meantime, more effort should go towards repurposing existing drugs by making TB antibiotic regimens and dosing modular so that treatment plans can be easily tuned for precise care.

We can no longer afford to take a one-size fits all approach to TB. It is our responsibility to leverage bench science advancement to improve patient outcomes *via* precision medicine.

## Author contributions

SS, SF, and QL contributed to the conceptual framework of the review. SS wrote the first draft of the manuscript and SF wrote additional sections. SS, SF, and QL completed edits and revisions. All authors contributed to the article and approved the submitted version.

## Funding

This study was funded by NIH/NIAID (P01AI132130, T32AI049928, T32AI132120).

## Conflict of interest

The authors declare that the research was conducted in the absence of any commercial or financial relationships that could be construed as a potential conflict of interest.

## Publisher's note

All claims expressed in this article are solely those of the authors and do not necessarily represent those of their affiliated organizations, or those of the publisher, the editors and the reviewers. Any product that may be evaluated in this article, or claim that may be made by its manufacturer, is not guaranteed or endorsed by the publisher.
